# Pediatric healthcare costs for patients with 22q11.2 deletion syndrome

**DOI:** 10.1002/mgg3.310

**Published:** 2017-08-12

**Authors:** Peter Benn, Sushma Iyengar, Terrence Blaine Crowley, Elaine H. Zackai, Evanette K. Burrows, Solomon Moshkevich, Donna M. McDonald‐McGinn, Kathleen E. Sullivan, Zachary Demko

**Affiliations:** ^1^ University of Connecticut Health Center Farmington Connecticut; ^2^ Natera, Inc. San Carlos California; ^3^ Children's Hospital of Philadelphia and the Perelman School of Medicine at the University of Pennsylvania Philadelphia Pennsylvania

**Keywords:** 22q11.2, healthcare costs, pediatric, prenatal screening

## Abstract

**Background:**

The 22q11.2 deletion syndrome is a variably expressed disorder that can include cardiac, palate, and other physical abnormalities, immunodeficiency, and hypocalcemia. Because of the extreme variability in phenotype, there has been no available estimate of the total medical expenditure associated with the average case.

**Methods:**

We have developed a model to estimate the cost from the time of diagnosis to age 20. Costs were based on patients seen at a specialty center but also considered those components of care expected to have been provided by external healthcare facilities. Expense was based on billed medical charges extracted from the electronic medical billing system for all patients with a diagnosis of DiGeorge or velocardiofacial syndrome from 1993–2015. Expenditures included maternal prenatal care directly related to an affected pregnancy, molecular/cytogenetic diagnosis, consultations, surgery, and/or other treatment and management. Most mental health services (except inpatient), therapy related to cognitive, behavioral, speech, pharmacy, and nonmedical costs (special education, vocational, respite, lost earnings) were not included.

**Results:**

Data were available for 642 patients with 50.7% diagnosed prenatally or in the first year of life. The average cost for a patient was $727,178. Costs were highest for patients ascertained prenatally ($2,599,955) or in the first year of life ($1,043,096), those with cardiac abnormalities or referred for cardiac evaluation ($751,535), and patients with low T‐cell counts ($1,382,222).

**Conclusion:**

This study demonstrates that there are significant medical costs associated with 22q11.2 deletion syndrome.

## Introduction

The most common clinically significant microdeletion in the human genome lies within a 3 Mb region on chromosome 22 and results in the “22q11.2 deletion syndrome” (Wou et al. 2016). This disorder has been previously known as DiGeorge syndrome, velocardiofacial syndrome, conotruncal anomaly face syndrome, autosomal dominant Opitz G/BBB syndrome, Sedlackova syndrome, or Cayler cardiofacial syndrome (McDonald‐McGinn et al. 1993). Although most patients have the standard LCR22A‐LCR22D deletion (or a variant atypical nested deletion that encompasses some of the same genes), a remarkable characteristic of this disorder is the considerable variability in both the types of abnormalities that may be present and their severity (McDonald‐McGinn et al. 2015). Some of the more common physical findings include cardiac defects, palatal abnormalities, immune deficiencies, hypocalcemia, gastrointestinal, skeletal, and renal anomalies (McDonald‐McGinn et al. 1993). Additionally, learning disabilities and other developmental delay is observed in 70–90% of patients, autism or autistic spectrum disorder is found in approximately 20% of children, and psychiatric illness (especially schizophrenia) is present in 25% of patients (Murphy et al. 1999; Schneider et al. 2014) Because of the diversity and variability in the condition, there is often a significant “diagnostic odyssey” before a definitive diagnosis through molecular cytogenetic testing is obtained. Although most cases of 22q11.2 arise as de novo mutations, in a minority of cases the deletion is inherited (McDonald‐McGinn et al. 2001; Poirsier et al. 2016). There are also rare cases where individuals have been diagnosed only as a result of ascertainment through a more severely affected family member (McDonald‐McGinn et al. 2001) and some patients are not diagnosed until adulthood (Furuya et al. 2015; McDonald‐McGinn et al. 2015).

Medical expenditures incurred for patients with 22q11.2 deletion syndrome reflect the highly variable phenotype. Evaluating overall costs can be important, particularly for the support of regional programs that provide comprehensive care for patients with 22q11.2 deletion syndrome. However, such cost assessments are difficult to carry out, particularly in the US healthcare system in which expenses can be incurred for numerous specialties with services based at multiple institutions.

In a previous report, we noted high pediatric healthcare charges for patients with 22q11.2 deletion syndrome who also had cardiac or immune issues but did not attempt to estimate average expenses (Sullivan et al. 2016). In this study, we have carried out a more comprehensive medical cost assessment for individuals with 22q11.2 deletion syndrome up to age 20 years. The modeling carried out takes into consideration care that was provided at a tertiary care center and also equivalent services provided at other medical centers. The methods used in the analysis may be appropriate for cost analysis for other disorders where data is incomplete due to fragmented healthcare service provision.

## Methods

### Ethical compliance

This study was reviewed and determined to be exempt from Institutional Review Board investigative study requirements.

Patients were identified through the electronic medical record at a single tertiary medical center, the Children's Hospital of Philadelphia (CHOP), where a specialty center for evaluation of patients with 22q11.2 deletion syndrome has been established. All patients had a diagnosis of chromosome 22q11.2 deletion syndrome based on ICD9 codes of 279.11 (DiGeorge syndrome) and 758.32 (velocardiofacial syndrome and 22q11.2 deletion syndrome) and were seen at CHOP between 1993 and 2015. Included in the dataset were information relating to the patient's age of diagnosis, the age at every visit, annual costs for every year of life in which the patient received clinical care at CHOP, whether the patient had cardiac abnormalities (Y/N) or a low T‐cell count (Y/N).

A further review of individual patient charts was carried out to confirm the diagnoses based on results from microarray analysis, fluorescence in‐situ hybridization, or multiplex ligation‐dependent probe amplification. Exclusion criteria included an unconfirmed diagnosis and an uncertain date of diagnosis, or cases in which the chromosome breakpoints were outside the usual A–D region of 22q11.2 and an atypical phenotype might therefore be expected (Shaikh et al. 2000; Hestand et al. 2016). The final dataset used in this analysis is a subset of data presented in an earlier publication (Sullivan et al. 2016).

Medical “costs” (USD) for each patient were extracted from the electronic medical billing record. Although these “costs” were actually billed charges rather than actual expenses, we assumed these charges were a reasonable proxy for costs since they are CPT–code‐related maximum recoverable fees. Annual costs incurred by each patient from the prenatal year to age 20 years, were stratified according to patient birth year, starting at the age at diagnosis. Prenatal costs related to the birth of an affected individual were included in that patient's medical costs. T‐cell counts were measured within a few months either side of 1 year of age, with a count of <700 cells/mm^3^ considered low.

Recognizing that data in this billing system was substantially incomplete due to patients receiving medical care for only a limited number of years within the CHOP medical system, we established a model that would adjust for missing data. We assumed that in postdiagnosis years in which care was not provided by CHOP, equivalent medical services at similar cost would be provided elsewhere. Therefore, we calculated the average annual healthcare billings for each group of patients with a particular age of visit in which care had been provided by CHOP, and weighted those averages by the cumulative percentage of patients who had been diagnosed by that year. The sum of these weighted average medical costs from diagnosis to age 20 years, provided a total average pediatric healthcare cost for patients diagnosed with 22q11.2 deletion syndrome. Specifically, for each year of life, we calculated an age‐specific cost by averaging the costs for all patients with a cost listed in the CHOP system for that year of life. We then calculated the number and percent of patients from the cohort who were diagnosed with the 22q11.2 microdeletion in each year of life, as well as the cumulative percent of patients who had been diagnosed by that year of life. Finally, to obtain the weighted average cost for each year of life, we multiplied the average age‐specific cost by the cumulative percentage of patients who had been diagnosed by that year of life. Summing the weighted average costs for each year of life, from the prenatal year through the 20th year, gives the overall average childhood hospitalization cost. Calculations of the average pediatric medical care costs for different subsets of patients were calculated the same way.

To assess whether inconsistencies in the data arising from a larger than average proportion of care being administered in the year of diagnosis were biasing the model, we considered an alternate model in which we treated costs incurred in the year of diagnosis as separate from other costs. Additionally, we considered a third model that included costs incurred in years prior to the year of diagnosis. In both cases, the estimated total average pediatric medical cost differed by no more than 5% from the estimate arrived at by the model described earlier, thus we did not present these findings here. To provide the most conservative (lower) estimate of the average cost, patients diagnosed with a 22q11.2 deletion after the age of 20 (and therefore had no recorded 22q11.2 deletion syndrome pediatric costs), were included in the denominator when calculating the average pediatric healthcare costs.

Similar cost analyses were also carried out for subsets of patients who had been diagnosed at different ages (prenatal year, birth year, 1–5 years, 6–10 years, 11–15 years, and 16–20 years), and for patients who had cardiac defects or low T‐cell counts. Finally, to evaluate whether there was under‐ascertainment of medical costs for patients living far from CHOP, we further analyzed the data with respect to the first three digits of the patient's postal ZIP code. This divided the dataset into those patients living within approximately 30 miles of CHOP and those residing more distally; total pediatric costs were calculated for both geographic subsets.

This study was approved by the Human Subjects Review Board at CHOP.

## Results

The study population consisted of 642 patients with 22q11.2 deletion syndrome. Of these, 325 (50.6%) patients were diagnosed prenatally or in their first year of life; the remainder had highly variable ages at diagnosis (Table 1). Clinical services were provided by CHOP in a total of 2451 patients‐years, of which 2366 were from the year of diagnosis or later; an average of 3.8 patient‐years per patient. Figure S1 shows the distribution of years for which clinical services were provided to patients with 22q11.2 deletion syndrome.

**Table 1:** Distribution of patients and average number of patient‐year data contributed, by age at diagnosis

Age at diagnosis	Patients, *n* (%)	Patient‐years, mean
Prenatal	30 (4.7)	4.3
YOB	295 (46.0)	4.4
1	51 (7.9)	4.0
2	52 (8.1)	4.0
3	43 (6.7)	3.6
4	32 (5.0)	3.5
5	26 (4.0)	2.7
6	21 (3.3)	4.2
7	11 (1.7)	3.9
8	9 (1.4)	3.4
9	13 (2.0)	2.0
10	8 (1.2)	1.9
11	6 (0.9)	4.2
12	4 (0.6)	1.8
13	6 (0.9)	2.3
14	2 (0.3)	0
15	1 (0.2)	3.0
16	4 (0.6)	3.5
17	1 (0.2)	2.0
18	4 (0.6)	2.0
19	2 (0.3)	1.0
20	1 (0.2)	0
21+	20 (3.1)	0
Total	642 (100)	

**Figure 1:**
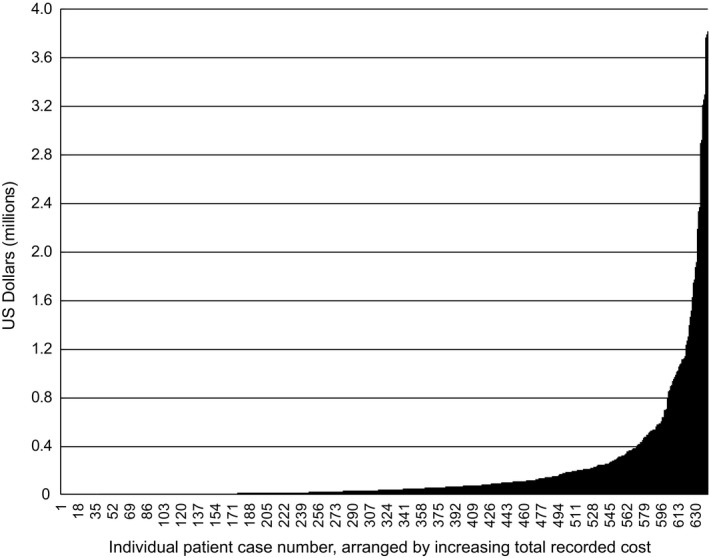
Distribution of individual patient costs.

Based on our model, which took into account patient‐years in which clinical service was not provided by CHOP, the average pediatric medical care cost associated with a diagnosis of 22q11.2 deletion syndrome in the general population was estimated to be $727,178 (calculations shown in Table 2). The costs calculated for specific subsets of patients are shown in Table 3. Costs were highest for patients ascertained prenatally or in the first year of life, presumably reflecting the fact that more severely affected cases are more likely to have come to attention early, and that they have a larger number of years of accumulated costs. For the patients that lived within 30 miles of CHOP (*n* = 257), the estimated costs were slightly higher ($775,321) compared to patients living more distally (*n* = 401; $698,151). Patients with cardiac abnormities or who were referred for cardiology examination (*n* = 601; 94.0%) had average costs of $751,535, which was substantially higher than the $143,805 for patients that did not receive a cardiology consult (*n* = 41; 6.0%). Similarly, for patients with low T‐cell counts (*n* = 43; 7.0%) the average costs amounted to $1,382,222, which was much higher than the costs for patients with normal T‐cell counts (*n* = 599; 93.0%, $652,287). The presence of specific clinical features was thought to contribute to the large discrepancy in per patient costs. For example, the 60 (9.3%) patients with highest costs (>$500,000 in total billing costs) accounted for over 65% of the total costs for all 642 patients (Fig. 1); all of these patients had cardiac abnormalities (vs. 94.0% for the full cohort) and 22.0% had an abnormal low T‐cell count (vs. 7.0% for the full cohort).

**Table 2:** Calculation of modeled average pediatric healthcare cost

Age of diagnosis or treatment	Treatment age‐specific average cost	Patient with costs forgiven treatment age	Patients with given Dx age, *n* (%)	Cumulative % patients diagnosed by given age	Weighted costs
Prenatal	$888,959	21	30 (4.7)	4.7	$41,540
YOB	$380,999	120	295 (46.0)	50.6	$192,873
1	$83,661	138	51(7.9)	58.6	$48,998
2	$52,835	144	5 (8.1)	66.7	$35,223
3	$27,891	147	43 (6.7)	73.4	$20,462
4	$26,277	154	32 (5.0)	78.4	$20,588
5	$32,592	159	26 (4.1)	82.4	$26,855
6	$23,059	168	21 (3.3)	85.7	$19,754
7	$25,811	137	11 (1.7)	87.4	$22,554
8	$25,534	143	9 (1.4)	88.8	$22,670
9	$36,213	134	13 (2.0)	90.8	$32,885
10	$19,054	131	8 (1.3)	92.1	$17,541
11	$17,582	118	6 (0.9)	93.0	$16,350
12	$16,321	108	4 (0.6)	93.6	$15,279
13	$24,428	110	6 (0.9)	94.6	$23,096
14	$23,450	109	2 (0.3)	94.9	$22,244
15	$24,386	94	1 (0.2)	95.0	$23,171
16	$21,677	84	4 (0.6)	95.6	$20,732
17	$27,937	85	1 (0.2)	95.8	$26,762
18	$35,597	60	4 (0.6)	96.4	$34,322
19	$21,501	44	2 (0.3)	96.7	$20,797
20	$23,204	46	1 (0.2)	96.9	$22,481
>20	N/A		20 (3.1)	100	
Total					$727,178

**Table 3:** Average childhood medical costs for the full cohort and defined subcohorts

Cohort category (*n*)	Average cost ($)
Full cohort (642)	727,178
Costs by age at Dx	
Prenatal (30)	2,599,955
Birth year (295)	1,043,096
Age 1–5 (204)	367,039
Age 6–10 (62)	263,775
Age 11–15 (19)	137,091
Age 16–20 (12)	92,052
Costs by distance from CHOP	
<30 miles (257)	775,321
>30 miles (401)	698,151
Costs by clinical phenotype	
Cardiac abnormality (601)	751,535
No cardiac abnormality (41)	143,805
Low T‐cell count (43)	1,382,222
Normal T‐cell count (599)	652,287

## Discussion

The clinical management of patients with 22q11.2 deletion syndrome requires diverse medical services (Bassett et al. 2011; Fung et al. 2015). These include medical genetics; multiple subspecialty consultations for the specific physical and behavioral symptoms associated with the condition; surgery for cardiac, palatal, and other birth defects; and additional management for various other organ, endocrine, and immune deficiencies. In this study, we assessed the average per‐patient childhood healthcare costs associated with these diverse medical services for patients up to the age of 20 years. The analysis recognizes that some of the most severely affected patients will be diagnosed in the neonatal period while for others, diagnosis and referral to a specialty referral center may occur much later. Because the data were extracted from a specialty referral center that receives some referrals of patients located far from the hospital, we analyzed costs separately by patients’ geographic location.

Based on data extracted for all patients included in this study, the average pediatric healthcare cost was estimated to be $727,178. However, there was considerable variation that reflects the wide phenotypic spectrum of 22q11.2 deletion syndrome. The cost estimates were highest for patients with a neonatal diagnosis, those with cardiac abnormalities or T‐cell deficiencies, and those living within approximately 30 miles of CHOP, who were expected to have received most of their care at CHOP. Very high costs were also noted for prenatally diagnosed cases, particularly in the first year. This reflects costs associated with prenatal ultrasound and ascertainment of cases with major fetal abnormalities that required early and expensive surgical interventions.

The study builds on a previous analysis of healthcare costs associated with this diagnosis in which it was shown that some children can have extraordinarily high healthcare costs, particularly when cardiac abnormalities and immunodeficiency were present (Sullivan et al. 2016). That study considered only the total direct billing charges recorded for each patient in the billing system from 1993–2015. Patients who received care earlier than 1993, or later than 2015, or who only received care for a limited interval at the CHOP, would not have had their total costs recorded. This study, which is based on a subset of the original data, but with added confirmation of diagnosis through individual review of each clinical chart, assesses total costs by considering cumulative average charges for each patient‐year from age at diagnosis to age 20 years, and adjusts for missing data.

A strength of the study is the large number of patients available for analysis. A weakness of the study is the assumption made in our model that for the postdiagnosis patient‐years with missing data, patients visited other health centers and received a comparable level of care at a similar cost. We assumed that all patients with 22q11.2 deletion syndrome received the recommended set of clinical management steps (Bassett et al. 2011; Fung et al. 2015). However, we recognize that our patient cohort consisted of referrals to a tertiary care facility, which could be weighted toward patients with more severe phenotypes. We attempted to correct for this bias by analyzing costs for the subset of patients located geographically close (<30 miles) to CHOP, for whom this care center might be a primary care facility for all affected children across the clinical spectrum. For patient‐years in which costs were incurred at CHOP, any additional medical expenses associated with care outside CHOP would not be captured in the model. Moreover, there may have been some less severely affected individuals who did not come to medical attention by age 20 years that were not included in the average costs. We did include 20 such cases but it remains possible that some additional cases were omitted. Another limitation of the study was that we did not consider costs incurred prior to the establishment of a formal diagnosis of 22q11.2 deletion syndrome (including diagnostic odyssey costs); neither could we separate out costs attributed to routine pregnancy management for the mothers of affected children who received care in the prenatal year, or costs related to studies on additional family members. We also assumed no pediatric mortality and did not subtract costs that would normally be incurred by unaffected children. All estimates of costs were based on billed charges; actual reimbursement to the medical center would be expected to be lower. No adjustment was made for time‐related inflation of costs.

There were also likely to be other significant medical costs that were not included in these analyses. Our available data is largely limited to the 0–20 year age range because the hospital focuses on services for children. There may also be some components of pediatric healthcare that were outside the scope of the hospital. This includes psychiatric services for which requirements for clinical services can be considerable (Schneider et al. 2014). Unfortunately, our de‐identified electronic database did not allow access to information on all neurological conditions (diagnosed or suspected autism, schizophrenia, behavioral problems, etc.) present in this cohort of patients. Moreover, many psychiatric illnesses associated with this condition have a later onset or require ongoing local treatment which would not be captured. Approximately 25% of patients with 22q11.2 deletion syndrome would be expected to receive a diagnosis of schizophrenia (Murphy et al. 1999) with unpredictable age for the prodromal and overt clinical phases. Per patient annual Medicare costs for the diagnosis of schizophrenia alone have been estimated to be nearly $8000 higher than that for a general Medicare population (Feldman et al. 2014). Other patients may not receive a formal diagnosis of schizophrenia but still require a substantial amount of psychiatric services due to a diagnosis of attention deficit hyperactivity disorder (ADHD), autism spectrum disorder (ASD), anxiety/mood disorder, or psychotic disorder (Schneider et al. 2014). Other additional medical costs for adults with the syndrome will reflect the diversity of the phenotype and extent of disability (McDonald‐McGinn et al. 1993). Based on survival studies in adults with the disorder, median age at death was 41.5 years (Bassett et al. 2009). Cumulative adult healthcare costs, especially long‐term residential care costs, can therefore be considerable.

Nonmedical costs were not addressed in this study, but they also constitute a substantial burden for families and society. One estimate for the average additional annual cost to a family for a child with a disability is $30,000 per year (Stabile and Allin 2012). Expenses associated with special education, assisted living and transportation costs have been estimated for individuals with autism and pervasive developmental disorders. For example, data from the California Department of Developmental Services suggests a societal annual cost of $10,500 for a child and $26,500 for an adult with autism (Leigh et al. 2016). Some of the nonmedical costs that were not considered in this analysis include long‐term speech and language therapy (Spruijt et al. 2014), cognitive therapy, physical therapy for hypotonia, behavioral counseling (Gerdes et al. 2001), occupational and vocational counseling, respite care, and lost wages. The combination of both physical and mental disability can be expected to compound costs (Horlin et al. 2014). Early diagnosis of 22q11.2 deletion syndrome together with development of an individualized plan for therapy and special education is advocated (Gerdes et al. 2001). The extreme diversity of the phenotype for 22q11.2 deletion syndrome precludes a complete estimate of a full lifetime average cost for this condition.

Considerable progress has been made in establishing prenatal (Helgeson et al. 2015; Wapner et al. 2015; Gross et al. 2016) and neonatal (Tomita‐Mitchell et al. 2010; Pretto et al. 2015) screening for 22q11.2 deletion syndrome. These approaches offer the possibility of relief from some of the medical and financial burden associated with this condition, especially costs related to the “diagnostic odyssey.” Early identification of affected individuals should result in better overall management (Bassett et al. 2011; McDonald‐McGinn et al. 2015) including earlier ascertainment and better management of cardiac defects (Chang et al. 2008), feeding difficulties and hypocalcemia (Bassett et al. 2011; McDonald‐McGinn et al. 2015), as well as avoidance of seizures that may improve long‐term physical and cognitive function (Cheung et al. 2014). In the meantime, our study demonstrates a diagnosis of 22q11.2 deletion syndrome is associated with a high cost to both families and society and there is a need for substantial financial support for their pediatric patient healthcare.

The approach to estimating the healthcare costs used in this study should be applicable to other genetic disorders where there are complex and highly variable phenotypes and where there are specialty referral clinics that provide comprehensive services. This includes some chromosome abnormalities, single gene disorders, and other multifactorial conditions. Greater understanding of costs could result in improved allocation of resources. Hopefully, earlier diagnoses for many of these disorders will ultimately decrease lifetime costs through improved patient management.

## Description of Supplemental Data

Supplemental data includes one figure in an Excel file.

## Conflict of Interest

ZPD and SM are employees of Natera, and PB is a paid consultant of Natera; all three hold shares or share options in the company. DMM has given lectures on 22q11.2 deletion syndrome for Natera.

## Supporting information


**Figure S1**. Graphical display of the available data for each treatment year (columns) for each patient (rows) for the entire cohort, ordered by year of diagnosis.
