# Supplementary material for: Pediatric healthcare costs for patients with 22q11.2 deletion syndrome
Source: Mol Genet Genomic Med. 2017 Aug 12;5(6):631–8. doi: 10.1002/mgg3.310 (PMC6234953; doi:10.1002/mgg3.310)
Supplement: Supplementary file 1 — Figure S1. Graphical display of the available data for each treatment year (columns) for each patient (rows) for the entire cohort, ordered by year of diagnosis. [file MGG3-5-631-s001.pdf]

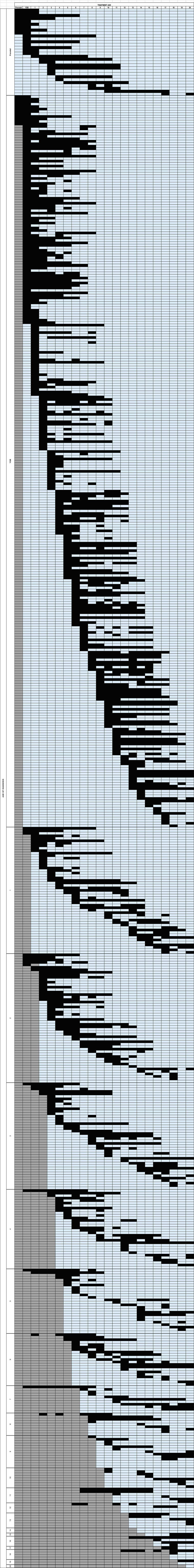
TRAETMENT AGE
Prenatal
YOB
1
2
3
4
5
6
7
8
9
10
11
12
13
14
15
16
17
18
19
20
AGE OF DIAGNOSIS
